# EVALUATING MHEALTH APPLICATIONS FOR OLDER ADULTS WITH DIABETES: SYSTEMATIC META-ANALYSIS OF CLINICAL TRIALS

**DOI:** 10.1093/geroni/igad104.3783

**Published:** 2023-12-21

**Authors:** Renato Ferreira Leitao Azevedo, Michael Varzino, Erika Steinman, Wendy Rogers

**Affiliations:** University of Illinois Urbana-Champaign, Urbana, Illinois, United States; University of Illinois Urbana-Champaign, Champaign, Illinois, United States; University of Illinois Urbana-Champaign, Champaign, Illinois, United States; University of Illinois Urbana-Champaign, Champaign, Illinois, United States

## Abstract

Diabetes is a common chronic condition among older adults and health technology interventions for diabetes management have the potential to support their self-management. However there is no systematic review and meta-analysis of the effects of mobile health applications (mHealth apps) for older adults. We conducted a systematic review and meta-analysis of randomized controlled trials (RCTs) to examine the effectiveness of mHealth apps for improving older adults’ diabetes outcomes. We searched the EBSCO, Clinical Trials, Cochrane Library, ProQuest, PubMed, ScienceDirect, Scopus, and Web of Science databases for relevant studies. We retrieved 4247 papers and two reviewers completed a full screen review of 257. We excluded papers if the study was not a RCT, did not examine the effect of mHealth apps, or was not conducted with older adults, resulting in 7 papers included in the final analysis. Our results indicate that mHealth apps can be effective for older adults managing diabetes, with significant reductions in glycated hemoglobin (HbA1C) (Hedge’s g: -0.40, 95% CI [-0.75 to -0.06]), and fasting blood sugar (FBS) levels (g: -0.29, 95% CI [-1.52 to 0.42]), as well as improvements in medication adherence (g: 0.93, 95% CI [0.53 to 1.28]). These effect sizes are comparable with other meta-analysis conducted across different aging groups. We describe each intervention and evaluate the risk of bias and the app components that could relate to effectiveness to inform the design of other diabetes management RCTs, and more broadly, the design of other mHealth tools for older adults. Pre-registration: http://doi.org/10.17605/OSF.IO/AWVCX

